# Ambulatory screening and decontamination to prevent *Staphylococcus aureus* complications in patients undergoing elective surgery (STAUfrei): study protocol for a controlled intervention study

**DOI:** 10.1186/s12879-020-4804-7

**Published:** 2020-01-31

**Authors:** Antonia Bauer, Martin Grünewald, Hans Eberhardt, Rieke Schulz, Peter Martus, Bernd Brüggenjürgen, Stefanie Joos, Heidrun Sturm

**Affiliations:** 1 Institute of General Practice and Interprofessional Care, Faculty of Medicine of the Eberhard Karls University Tübingen, University Hospital Tübingen, Osianderstraße 5, 72076 Tübingen, Germany; 2 Klinikum Heidenheim, Heidenheim, Germany; 3 Pathways Public Health GmbH, Berlin, Germany; 4 Institute for Clinical Epidemiology and Applied Biometry, University Hospital Tübingen, Tübingen, Germany; 5 Steinbeis Business Academy, Berlin, Germany

**Keywords:** *Staphylococcus aureus*, Decontamination, Prevention, Surgical site infection, MRSA, MSSA, Intersectoral care

## Abstract

**Background:**

Surgical site infections (SSI) are the most common health care associated infections in German acute hospitals and can result in prolonged hospital stays, increased use of antibiotics and utilisation of care. *Staphylococcus aureus* bacteria (methicillin-resistant S Aureus (MRSA) and methicillin-susceptible S Aureus (MSSA)) are amongst the most prominent causes of SSI. While up to 90% of documented S Aureus colonization is already detectable prior to hospital admission, the majority of hygiene measures in Germany is focused on the hospital setting. It is hypothesized that early detection and decontamination of S Aureus colonization in primary care can prevent health care associated infections and reduce the number of S Aureus isolates in the hospital setting.

**Methods:**

This study is a controlled interventional study (*N* = 13,260) with a pre-post comparison. The intersectoral intervention (over 2 years) will encompass the following elements: ambulatory detection and decontamination of MRSA and MSSA prior to elective surgery combined with a structured follow-up care. Patients from the control group will be screened in the hospital setting, in accordance with the standard operating procedure (SOP) in routine care. The primary endpoint is the reduction of MRSA and MSSA colonization upon hospital admission. Secondary endpoints are complication rate (SSI), length of stay, recolonization of patients (3 and 6 months after release), patient and provider satisfaction, patient compliance and cost development.

**Discussion:**

In case of positive results, the chance of a widespread uptake and implementation in routine care are considered high. The active involvement of primary care providers in the implementation of screening and decontamination as well as follow-up care is a unique feature of this study. The positive resonance of primary care providers during the recruitment phase highlights the relevance of the topic to the participating actors. These efforts are coupled with patient education and specifically trained medical staff, promising a sustained impact. The STAUfrei care pathway can homogenize current practices in routine care and provide a template for further intersectoral cooperation.

**Trial registration:**

German Clinical Trials Register (DRKS), DRKS00016615. Registered on April 1st, 2019.

## Background

*Staphylococcus aureus* (S Aureus) bacteria are detectable on the skin or mucus membranes of the nasal passage and pharynx in about 20 to 30% of the population [1]. Most of S Aureus isolates exhibit sensitivity to beta-lactam antibiotics (e.g. Methicillin or Oxacillin) and are therefore referred to as methicillin-sensitive S Aureus (MSSA). In contrast, methicillin-resistant S Aureus (MRSA) bacteria are resistant to these antibiotics. MRSA isolates are much rarer than MSSA, only affecting about 1 to 2% of the population in Germany [2].

During surgical interventions both bacterial strains (MSSA and MRSA) can cause infections as they gain access to other organs or tissues. If the infection occurs up to 30 days post-surgery and affects the incision or deep tissue at operating sites they are termed surgical site infections (SSI) [3]. Surgical site infections remain the second most common health-care associated infection (HAI) in Europe and the US [4]. SSIs are associated with prolonged hospital stays, additional physician visits, impairment of the healing process and elevated use of antibiotics [5]. The national guideline by the Robert Koch Institute (RKI) [6] on the prevention of SSI, recommends establishing screening and decontamination procedures for S Aureus, in particular for high risk surgical interventions. While MSSA is increasingly recognized as a risk factor internationally, evidence on the effectiveness of screening and decontamination protocols for MSSA is scarce and it is often not explicitly addressed in national guidelines [5, 6]. Correspondingly, preoperative screening and decontamination efforts in German routine care are targeted at MRSA only. Evidence suggests that decolonization regimes for MSSA can be protective against SSI, particularly since MSSA is a noteworthy contributor to SSI prevalence rates [7, 8].

Unlike for MSSA; the evidence base on MRSA decolonization is more extensive. While the establishment of hospital based screening, isolation and decolonization routines may have contributed to a general decline of MRSA, Germany is still lagging behind when compared internationally [6, 9, 10]. The effectiveness and efficiency of universal screening regimes for MRSA have been documented in several studies, yet screening measures in German routine care are strictly risk based [11–13]. This may result in colonized patients without a corresponding risk profile to remain undetected. Furthermore, decolonization and hygiene measures are commonly implemented during an inpatient hospital stay [7, 11, 12, 14, 15]. With up to 90% of S Aureus cases already being detectable prior to hospital admission [16], shifting screening and decontamination to the primary care setting can provide an effective tool to prevent the introduction of MRSA to the hospital setting. To date only a limited number of studies have assessed the effectiveness of MRSA decontamination regimes in the home environment [14, 17]. Findings point to the importance of patient education and self-sufficiency as integral parts of successful decolonization as direct clinical supervision is lacking in the home setting [14, 17, 18]. Next to that, post-discharge follow-up and decontamination measures can lower the risk of SSIs for MRSA carriers as well as rehospitalisation [19]. In spite of the evidence, the absence of remuneration options has at least partly hampered a systematic prevention and treatment of MRSA colonization and associated complications (SSI) in Germany.

STAUfrei aims to fill these treatment gaps by implementing an intersectoral care pathway stretching from ambulatory screening and decontamination (independent of risk profile) to a structured follow-up care in the primary care setting. Remuneration options for preventative (screening and decontamination) and follow-up (re-)colonization and infection control) care in the outpatient setting is offered by the major health insurance funds in the district. In order to investigate possible advantages of this novel care pathway compared to the current standard operating procedure in routine care, the latter was chosen as a comparison. This is also linked to the overarching aim of a potential nation-wide implementation.

## Methods/design

### Aim

The primary aim of the study is to reduce cases of MRSA and MSSA colonization upon hospital admission. Secondary objectives include prevention of postoperative complications (SSI) and recolonization through a structured follow-up care in the outpatient setting. Additionally, patient compliance, patient and provider satisfaction as well as cost developments will be evaluated.

In order to evaluate the effect of the intervention on a clinical and district level, we aim to carry out a time-series analysis with a pre-post comparison (see Table 1). On a clinical level routine data from the intervention phase (2019–2020) will be contrasted with historic routine data (2015–2018) from the local community hospital. The effect of the intervention will be evaluated both, in terms of clinical and health economic parameters. The district level analysis will be based on routine data from the health insurance fund (AOK Baden-Württemberg). The aim is to evaluate whether the (potential) positive trend in Heidenheim is also observable in other districts. Control districts will be matched according to socioeconomic and demographic aspects (e.g. number and size of hospitals in district, age structure, unemployment rate).

**Table 1:** Primary and secondary objectives

Primary research question	
Does the implementation of measures in the primary care setting including ambulatory screening and decontamination reduce MRSA and MSSA colonization rates upon hospital admission?	
Secondary objectives	
Do primary care measures (patient supervision and support through GP practices pre- and post-surgery) reduce recolonization rates (MRSA) amongst patients who underwent elective surgery?	
Does a reduction in MSSA colonization upon hospital admission correlate with an improved quality of care measured by a reduction in surgical site infections, length of stay and rehospitalisation?	
Does the implementation of decontamination measures in the primary care setting lead to a reduction of associated costs for patients undergoing elective surgery?	
How is the feasibility of the intervention evaluated by participating GP practices?	
How do patients evaluate the feasibility of the decontamination in the home environment?	

### Study design

**Fig. 1:**
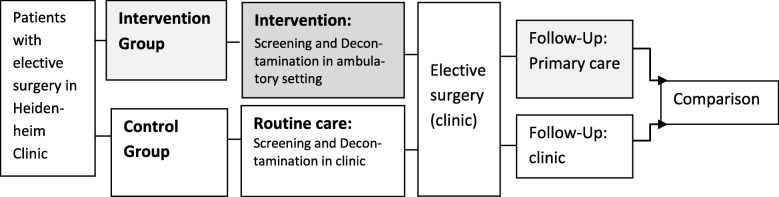
(A) Evaluation scheme for comparison of control and intervention group from 2019 onwards

In order to achieve these objectives the study will employ a controlled study design (A) with a time series analysis (pre-post comparison) on clinic and district level (B) (Fig. 1, Table 2). There is no randomization on practice or patient level. A detailed overview of the endpoints for analysis (A) and (B) can be found in under ‘Outcomes’.

**Table 2:** (B) Evaluation scheme for time series analysis on clinical (1) and district level (2) from 2015 onwards

	Control phase (Routine care)				Intervention Phase		
	T1 (2015)	T2 (2016)	T3 (2017)	T4 (2018)	T5 (2019)	T6 (2020)	T7 (2021)
(1) Clinical level based on clinical routine data							
Local Clinic	X	X	X	X	X	X	X
(2) District level based on health insurance data							
HDH^a^ District	X	X	X	X	X	X	–
Control District A	X	X	X	X	X	X	–
Control District B	X	X	X	X	X	X	–
Control District C	X	X	X	X	X	X	–

^a^*HDH* Heidenheim

It will not be possible to include the year 2021 in the analysis on district level based on health insurance data (see Table 1). This is due to a time lag in the data delivery of approximately 9 months at the respective insurance fund (time needed for internal quality assurance process).

### Setting

The study will be carried out in the local clinic in Heidenheim, Baden-Wuerttemberg. Heidenheim has a population of roughly 130,500 inhabitants distributed across elven cities and communities. Being the only hospital in the district, the local community hospital offers treatment to around 90% of local inpatient cases. The largest health insurance companies in the Heidenheim district are the AOK Baden Württemberg (Allgemeine Ortskrankenkasse), AOK-Bayern, BKK (Betriebskrankenkasse) and the IKK (Innungskrankenkasse). All have signed selective agreements (§140SGBV) with the local community hospital allowing the majority of the local population to partake in the study.

### Existing MRE-prevention activities in Germany

In Germany, primary and secondary care providers have established so-called MRE-Networks in order to limit the spread of multi-drug resistant pathogens (MRE). There are currently 150 MRE-Networks across Germany, with every federal district (Bundesland) having at least one major MRE-Network [20]. In Baden-Wuerttemberg nearly every regional district has an established MRE-Network. The local health authorities are responsible for coordinating regular meetings between participating care providers across all health care sectors (outpatient and inpatient care). Activities include the implementation of national guidelines developed by the Robert-Koch-Institute, the support of intersectoral, MRE-based information exchange, screening of patients at risk of colonization, decontamination during or after an inpatient stay, education for patients and care providers as well as to strengthen the cooperation between health care providers and local health authorities.

### Recruitment

Patients will be allocated to either study group based on their GPs. GP practices will be predefined as either control or intervention practice based on their participation in the regional MRE-Network. The intervention group will only contain practices that partake in the MRE-Network Heidenheim, while the control group will include non-participating practices from Heidenheim as well as other districts. With the engagement of the physicians association Heidenheim (Kreisärzteschaft Heidenheim) in the conception of the project, we expect a high acceptance amongst GPs. The recruitment of practices is jointly carried out by the Association of Statutory Health Insurance Physicians Baden-Wuerttemberg (Kassenärztliche Vereinigung Baden-Württemberg) and the physician association Heidenheim.

**Fig. 2:**
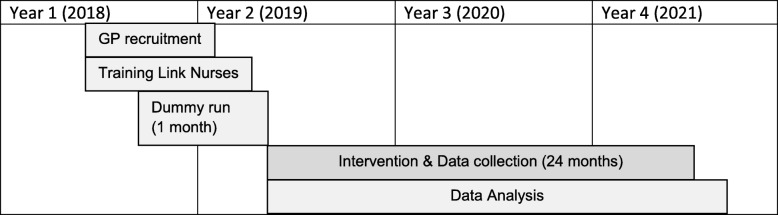
Timeline

The intervention phase will stretch over a period of 24 months and is preceded by a 6 months recruitment and preparation phase (also see Fig. 2).

### Participants

#### Inclusion and exclusion criteria

Patients aged 18 and above and scheduled for an elective procedures in the local community hospital are eligible for inclusion. Elective interventions from general surgery, accident surgery, urology and gynaecology as well as surgeries at high risk for infections such as installations of drainages, PEG tubes (percutaneous endoscopic gastrostomy), and stents will be considered. Yet only patients whose insurance companies agreed to partake in the study by signing selective agreements can be considered in the recruitment process. In the German Healthcare System, such agreements enable care outside the predefined care catalogues. Emergency patients are excluded from the study.

#### Sample size

**Fig. 3:**
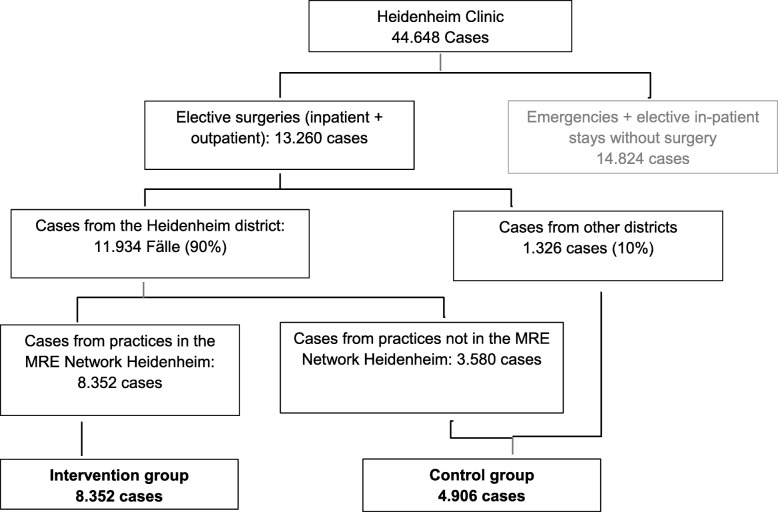
Sample size: intervention and control group (over 2 years)

The sample size was calculated to secure enough power for examining the effect of the intervention for both subgroups (MSSA and MRSA) on colonization status upon hospital admission (primary endpoint). We expect cluster effects to be relatively small as the introduction to decontamination measures will be carried out by link nurses who have received a standardized training. With the expected 13,260 Patients a power of 92% would be achievable, assuming that there are no cluster effects. Although the occurrence of a cluster effect cannot be excluded, we believe that with an eligible patient population of 13,260, potential reductions in power would still fall within an acceptable range (also see Fig. 3).

#### Patient pathway

##### (1) **Study inclusion and risk assessment** (intervention and control group)

**Fig. 4:**
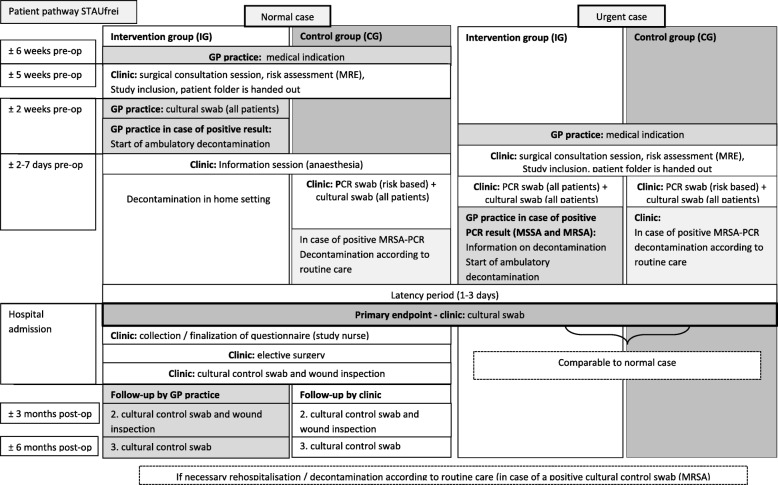
Patient pathway: intervention and control group

All patients, irrespective of risk of S Aureus colonization will be screened for risk of colonization with MRSA according to criteria defined by the Robert-Koch Institute (RKI) in the medical consultation session roughly 5 weeks prior to hospital admission (also see Fig. 4).

##### (2) **Early testing and decontamination** (intervention group only)

Patients in the intervention group will be tested for MRSA and MSSA by specifically trained medical assistants (so-called ‘Link Nurses’) in the GP practice approx. two weeks prior to surgery. Cultural specimens of both nares, the throat and any wounds will be sent to the central laboratory of the University Hospital Tübingen in order to achieve a uniform quality and thus comparability of test results. In normal cases there is sufficient time to await cultural test results before initiating decontamination. In case of urgency (time to surgery is less than 10 days) rapid preoperative screening of patients will be performed during the medical consultation session with the use of polymerase chain reaction-based diagnostic tests (PCR). The initiation of subsequent decontamination measures in the outpatient setting will be based on results from PCR swabs. Due to the high false-positive rates of PCR Test results, all swabs will also be tested culturally in the central laboratory in Tübingen [5]. Prevalence estimates will be based on cultural swabs only.

MRSA and MSSA positive patients will be instructed on decolonization measures by Link-Nurses at the respective GP practise. Decontamination measures will be carried out across a 5-day period by patients themselves in the home environment. Patients are asked to use an antibacterial wash lotion on a daily basis, nasal mupirocin ointment (3x per day), antiseptic mouth rinse (3x per day) as well as disinfection of commonly used objects as well as the living environment (daily). The decontamination regime for MRSA is in accordance with the most recent recommendations by the Robert-Koch Institute [5]. The decontamination regime for MSSA was largely adapted from MRSA. As a MSSA specific national recommendation is lacking, the decontamination protocol was created in accordance with relevant international publications and expertise of clinical hygienists [4, 7, 21, 22].

##### **(3) Hospital admission** (intervention and control group)

Upon admission all patients scheduled for elective surgery from both control and intervention groups receive a cultural swab of nares, throat and any wounds in order to assess the success of the decontamination (primary endpoint). In case the test result is positive, decontamination measures will be carried out in the hospital setting in accordance with the standard procedure in routine care.

##### (4) **Follow-up** (intervention group and control group)

Patients in the intervention group follow-up care will be provided by the GP, control patients will schedule follow-up appointments in the local community hospital.

- Surveillance of surgical site infections (SSI) within 90 days after hospital discharge
- Control swabs 3 and 6 months post discharge. The respective GPs will reach out to patients
- At follow-up visits Link Nurses provide patients with information on how to prevent recolonization
- In case of a positive test result for MRSA, decontamination according to routine care will be initiated

The modification of an allocated intervention is to be avoided. In some cases, however, the total of 5 days of decontamination may not be implemented for instance in case of urgency. This will not result in an exclusion of the specific patient in question. Change of study arm is not permitted during the study (intention to treat).

### Adherence to intervention

Training modules for outpatient nurses and doctors are expected to play a fundamental role in improving adherence of medical staff in practices to the intervention protocols. Training will be carried out by the consortium lead, a member of the public health department in Heidenheim as well as the lead of the physician association in Heidenheim. They will be supplemented by monthly case conferences /seminars hosted by the study team of the Heidenheim Clinic as well as personal site visits by the lead study nurse to all practices in order to provide individualized support. Moreover a support system will be established comprising an email service and a study hotline which can be used by both patients and providers. Inpatient processes will be under direct supervision of the internal study team in the Heidenheim clinic.

Patients who can decide to discontinue their participation in the study at any point in time. As compliance is also a relevant endpoint in this study, discontinuation of the decontamination process (i.e. due to related side effects, lack of motivation) without wishing to be excluded from the study itself, will not lead to exclusion from the analysis (primary analysis as intention to treat). Adherence to the intervention will be closely monitored via a decontamination schedule, where patients are required to tick off all completed decontamination steps. Patients may call link nurses in the GP practices for further information. A direct clinical supervision is not possible as the decontamination is self-administered in the home setting.

### Adverse events

All adverse events related to the intervention that lead to a discontinuation of the study will be documented by study personnel in the Heidenheim clinic. Reasons for discontinuation of the decontamination procedure will be collected via patient questionnaires. Adverse events directly related to the intervention (i.e. reaction of the skin, allergic reactions) are, however, expected to be rare.

### Outcomes

Colonization status (MRSA and MSSA) upon hospital admission will serve as the primary endpoint (see Table 3). Secondary endpoints include comparison of length of stay, recolonization and rehospitalisation as well as wound infection rates (SSI). In addition, associated costs of secondary care will be compared. Feasibility of decontamination measures and patient satisfaction with the intervention will be assessed via paper based questionnaires (patients) as well as focus groups (practice teams). In total we aim for 3–4 focus groups with 8–12 participants each. All focus groups will be recorded and subsequently transcribed. As clinical study staff is closely monitoring the implementation at primary care practices and is responsible for the overall project management, 1–2 focus groups with 4–6 participants will be conducted with this particular group. Feasibility of the intervention will also be assessed based on qualitative data (questions from providers and patients to the study coordination office) anonymously collected during the intervention period via the study hotline.

**Table 3:** Primary and secondary endpoints, data sources und collection methods

Primary data collection			Routine data collection Heidenheim Clinic	Routine data collection Health insurance data
Patients	GPs, Link Nurses	Clinical data	Clinical data*	Routine data AOK-BW**
• Acceptance / feasibility / satisfaction (QS) • Compliance (QS)	• Acceptance/ feasibility / satisfaction (FG)	• Primary endpoint: Colonization upon hospital admission (MSSA und MRSA) (Lab) • Wound infection rates (SSI) • Recolonization with MRSA 3 + 6 months post-surgery (Lab) • Risk assessment	• MRSA colonization rates (2015–2018) • Length of stay • Rehospitalisation rates • Costs secondary care (incremental cost monitoring)	• MRSA colonization rates (2015 onwards) • Wound infection rates (SSI) • Rehospitalisation rates • Costs primary and secondary care (incremental cost monitoring)

*QS* Questionnaires, *FG* Focus groups, *Lab* Laboratory test result, (A) data for study group comparison (IG vs. CG) and time series analysis* (B) data for time series analysis only**

### Data collection and management

A pooled routine dataset is sent from the AOK Baden-Wuerttemberg (health insurance fund) as well as the Klinikum Heidenheim to the Institute for Clinical Epidemiology and Applied Biometry (IKEaB, University Hospital Tübingen). The IKEaB will deliver extracts of the pseudonymized patient dataset to the Insitute of Gerneral Medicine and Interprofessional Care (IAIV, University Hospital Tübingen) and the Steinbeis Business Academy for statistical analysis. Data access will be granted upon written requests of the evaluators (IAIV and Steinbeis Business Academy). All of these transfers will be carried out using a secure, encrypted protocol. The relevant data sources are subject to quality management processes. Primary data (i.e. questionnaire data as well as the lab results, wound findings) will be stored and entered manually by study personal of the Heidenheim clinic via an electronic Case Report Form (eCRF, Koordobas©) provided by the IKEaB. All paper-based data collection forms will be securely stored in the study centre of the Heidenheim clinic. Manually entered data will be subject to plausibility checks carried out by the IAIV. Audio transcripts from focus groups will be stored electronically in a secure folder at the IAIV. Only study personnel have access to this folder. There will be no paper based versions of the transcripts. After the end of the study, all paper and electronic documents will be stored for 10 years and subsequently deleted.

### Statistical analysis

**The primary endpoint** (comparison of colonization rates prior to surgery) will be analysed separately for MRSA and MSSA using logistic regression modelling with GEE (Generalized Estimation Equation) correction to account for potential cluster effects. The power calculation (see section 2) was based on the commonly used significance levels for two-sided chi-square tests. Given the higher prevalence of MSSA, two different levels of significance were chosen for MSSA (0.001) and MRSA (0.049). **Analysis of secondary endpoints** will involve adjustment for covariates such as age and gender using logistic regression modelling with GEE correction. However, the interpretation of local significances is not designed to be strictly confirmatory. Due to the multitude of secondary parameters and the lack of a hierarchical structure, a correction for multiple tests is not necessary. Descriptive analyses are performed following scaling and observed data distribution.

**Qualitative data** from focus groups will be transcribed and then analysed using qualitative content analysis [23]. Data collection will continue until thematic saturation is reached. The resulting category system will help to understand the feasibility of the intervention in daily practice from the perspective of providers in outpatient and hospital care. It will thereby provide additional information to the patient perspective evaluated via questionnaire data. Additionally, all qualitative data systematically collected or observed during the study will be considered as data. This means that not only information from focus groups and questionnaires will be analysed, but also all data documented during hotline calls or observed during site visits. This data will be collected by study personnel in the Heidenheim Clinic via a logbook. Entries of free text will be categorized thematically. Data entries will then be analysed descriptively following observed data distribution. Quantitative analysis will be carried out in Microsoft Excel (Version 2010) und IBM SPSS Statistics (Version 25). For qualitative data analysis MAXQDA (Version 2018) will be used.

**Subgroup analysis** is planned for patients from gynaecology as the criteria for initiating a decontamination procedure are mostly based on the recommendation of the supervising physician. Furthermore, the primary goal regarding this subgroup analysis is to extract prevalence rates – the success of the decontamination will most likely not be the main focus of the analysis. This is also linked to the small number of patients expected to undergo a decontamination procedure. All patients who are not undergoing a surgical procedure will be considered as a potential subgroup. This is due to the possibility of slight variation in the patient pathway as well as a different risk of complications upon follow-up. All other stratifications will be considered in the statistical model. The robustness of primary and secondary findings will be assessed through sensitivity analysis. Depending on the proportion of missing values, we will either use imputation or only complete data-sets.

### Data monitoring

Regular data monitoring will be carried out by the study team at the Heidenheim Clinic. The study team is thereby independent from the sponsor, and have no competing interest. Data monitoring will only be carried out for data that is manually entered. Based on interim reports are performed every 3 months, the sponsor (German Federal Joint Committee, G-BA) can decide on the early termination of the project. Moreover, the steering committee meets every month, to deal with any relevant project issues.

### Composition of steering committee

The core committee is composed of a member from the University Hospital Tübingen, the AOK Baden-Württemberg (sickness fund), the head of the physicians association of Heidenheim, a representative of the public health department of Heidenheim as well as a representative from the BKK-Süd (sickness fund). Additionally, at least one member of the external project management company (Pathways Public Health GmbH) is taking part in the telephone conferences to support the consortium leadership in the project management. Besides the consortium leadership (Klinikum Heidenheim), at least one representative from each body within the STAUfrei consortium is taking part in monthly telephone conferences to monitor achievements and set-backs in the respective sub-project. Depending on the subject at hand, additional project members can be recruited to join telephone conferences.

## Discussion

We believe that the intersectoral care pathway can make a substantial contribution to the overall quality of care. Initially, outpatient costs may rise as more GPs are implementing screening and decontamination measures on a larger body of patients (not only risk based). Yet, these additional costs can potentially be compensated by cost reductions in secondary care. Decontamination measures will predominantly be implemented in the primary care setting thereby potentially reducing costs for prolonged hospital stays. Furthermore, a potential reduction in post-operative complications (e.g. SSI) can reduce costs for follow-up treatment. In the long term, an overall reduction in the microbial load can benefit all patients treated in the Heidenheim clinic. Taking into account the multifaceted benefits on patient and system level, we believe that measures implemented in the context of this study have a high potential of being integrated to routine care.

To date there is no systematic management of problematic bacteria in the German health care setting. Beyond this, an infrastructure enabling intersectoral cooperation is largely missing. By filling these gaps, STAUfrei has the potential to pave the way for a successful management of problematic bacteria (beyond S Aureus) in the future. A unique feature is the active involvement of primary care providers in the implementation of screening and decontamination measures as well as follow-up care. These efforts coupled with patient education and specific training for medical staff, promise a sustained impact. Finally, ambulatory screening and decontamination measures are provided to both MSSA and MRSA patients, thereby also recognizing MSSA as a risk factor for SSI. Even though MSSA is one of the most common causes of SSI, there are no national recommendations or remuneration options for screening and decontamination measures in the German context - the current routine practice is focussed on MRSA only. In case of positive results, screening and decontamination as well as corresponding remuneration options could be extended to also include MSSA. An evaluation of the acceptance and feasibility of the intervention will identify relevant barriers and enablers for a potential widespread implementation. The overarching aim of STAUfrei is to provide a template for other intersectoral cooperations, especially regarding other types of multidrug resistant bacteria (e.g. 4MRGN, 3 MRGN, VRE).

There is a risk of selection bias due to the recruitment of intervention practices via the MRE-Network Heidenheim. Additionally, the local community hospital has been active in MRE-prevention prior to the intervention onset. In order to identify potential (positive) effects of prior efforts, we carry out a time series analysis on a hospital and district level. The successful implementation of patient pathways within the hospital setting has proven to be partly dependent on departmental particularities (i.e. staffing, leadership). Current efforts are made to homogenize processes across all relevant departments. Assessing the feasibility of the novel care pathway in the clinical and outpatient setting will therefore take a central role in the overall evaluation.

## Data Availability

The datasets used and/or analysed during the current study are available from the corresponding author on reasonable request. Participant or patient data will be de-identified.

## Abbreviations

3 MRGN: Multi-resistant Gram-negative bacteria (resistant against 3 antibiotic groups)
4 MRGN: Multi-resistant Gram-negative bacteria (resistant against 4 antibiotic groups)
AOK: Allgemeine Ortskrankenkasse
GEE: Generalized Estimation Eq.
GP: General Practitioner
HAI: Health-care associated infections
MRE: Multiresistente Erreger (multidrug resistant pathogens)
MRSA: Methicillin-resistant S Aureus
MSSA: Methicillin-susceptible S Aureus
PCR: Polymerase chain reaction-based diagnostic test
PEG: Percutaneous endoscopic gastrostomy
RKI: Robert-Koch Institute
S Aureus: *Staphylococcus aureus*
SOP: Standard operating procedure
SSI: Surgical site infections
VRE: Vancomycin-resistant enterococci

## References

[1] Schrauder A WC. MRSA - Welche Maßnahmen sind nötig? Arzneiverordnung in der Praxis. 2016;43(2):70–74.

[2] Köck R, Mellmann A, Schaumburg F, Friedrich AW, Kipp F, Becker K (2011). The epidemiology of methicillin-resistant Staphylococcus aureus (MRSA) in Germany. Deutsches Arzteblatt International.

[3] Owens CD, Stoessel K (2008). Surgical site infections: epidemiology, microbiology and prevention. J Hosp Infect..

[4] World Health Organisation (WHO) (2016). Global Guidelines for the Prevention of Surgical Site Infection.

[5] Ruscher C (2014). Empfehlungen zur Prävention und Kontrolle von Methicillin-resistenten Staphylococcus aureus-Stämmen (MRSA) in medizinischen und pflegerischen Einrichtungen. Bundesgesundheitsblatt.

[6] Bundesgesundheitsblatt (2018). Prävention postoperativer Wundinfektionen %. J Bundesgesundheitsblatt - Gesundheitsforschung - Gesundheitsschutz.

[7] Schweizer M, Perencevich E, McDanel J, Carson J, Formanek M, Hafner J (2013). Effectiveness of a bundled intervention of decolonization and prophylaxis to decrease gram positive surgical site infections after cardiac or orthopedic surgery: systematic review and meta-analysis. BMJ..

[8] Edmiston CE, Ledeboer NA, Buchan BW, Spencer M, Seabrook GR, Leaper D (2016). Is staphylococcal screening and suppression an effective interventional strategy for reduction of surgical site infection?. Surg Infect.

[9] Kock R, Winner K, Schaumburg F, Jurke A, Rossen JW, Friedrich AW (2014). Admission prevalence and acquisition of nasal carriage of meticillin-resistant Staphylococcus aureus (MRSA) in German rehabilitation centres. J Hosp Infect..

[10] Wertheim HFL, Vos MC, Boelens HAM, Voss A, Vandenbroucke-Grauls CMJE, Meester MHM (2004). Low prevalence of methicillin-resistant Staphylococcus aureus (MRSA) at hospital admission in the Netherlands: the value of search and destroy and restrictive antibiotic use. J Hosp Infect..

[11] Lucet J-C, Paoletti X, Lolom I, Paugam-Burtz C, Trouillet J-L, Timsit J-F (2005). Successful long-term program for controlling methicillin-resistant Staphylococcus aureus in intensive care units. Intensive Care Med.

[12] Jog S, Cunningham R, Cooper S, Wallis M, Marchbank A, Vasco-Knight P (2008). Impact of preoperative screening for meticillin-resistant Staphylococcus aureus by real-time polymerase chain reaction in patients undergoing cardiac surgery. J Hosp Infect..

[13] Reilly JS, Stewart S, Christie P, Allardice GM, Stari T, Matheson A (2012). Universal screening for meticillin-resistant Staphylococcus aureus in acute care: risk factors and outcome from a multicentre study. J Hosp Infect.

[14] Humphreys H, Becker K, Dohmen PM, Petrosillo N, Spencer M, van Rijen M (2016). Staphylococcus aureus and surgical site infections: benefits of screening and decolonization before surgery. J Hosp Infect.

[15] George S, Leasure AR, Horstmanshof D (2016). Effectiveness of decolonization with Chlorhexidine and Mupirocin in reducing surgical site infections: a systematic review. Dimens Crit Care Nurs.

[16] (NRZ) NRfSvnI (2016). Modul MRSA-KISS Referenzdaten Berlin: NRZ.

[17] Ramos N, Skeete F, Haas JP, Hutzler L, Slover J, Phillips M (2011). Surgical site infection prevention initiative - patient attitude and compliance. Bull NYU Hosp Jt Dis.

[18] Lepelletier D, Saliou P, Lefebvre A, Lucet JC, Grandbastien B, Bruyere F (2014). “Preoperative risk management: strategy for Staphylococcus aureus preoperative decolonization” (2013 update). French society of hospital hygiene. Med Mal Infect.

[19] Huang SS, Singh R, McKinnell JA, Park S, Gombosev A, Eells SJ (2019). Decolonization to reduce Postdischarge infection risk among MRSA carriers. N Engl J Med.

[20] Robert Koch Institut (2017). MRE-Netzwerke in Deutschland.

[21] Higgins M, Bommireddy R, Shivji F, Al-Shukri J, Billson J (2018). Impact of MSSA screening on rates of surgical site infection following lumbar spine surgery. Eur Spine J.

[22] Smith H, Borchard K, Cherian P, Tai Y, Vinciullo C (2019). Randomized controlled trial of preoperative topical decolonization to reduce surgical site infection for Staphylococcus aureus nasal swab-negative Mohs micrographic surgery patients. Dermatol Surg.

[23] Schreier M (2014). Varianten qualitativer Inhaltsanalyse: Ein Wegweiser im Dickicht der Begrifflichkeiten. Forum Qualitative Sozialforschung.

